# Globally suitable areas for *Lycorma delicatula* based on an optimized Maxent model

**DOI:** 10.1002/ece3.70252

**Published:** 2024-09-20

**Authors:** Zhengxue Zhao, Lin Yang, Xiangsheng Chen

**Affiliations:** ^1^ Institute of Entomology Guizhou University Guiyang China; ^2^ Provincial Special Key Laboratory for Development and Utilization of Insect Resources of Guizhou Guizhou University Guiyang China; ^3^ Guizhou Key Laboratory for Agricultural Pest Management of Mountainous Region Guizhou University Guiyang China; ^4^ College of Agriculture Anshun University Anshun China

**Keywords:** climate change, invasive pest, *Lycorma delicatula*, Maxent model, pest management, suitable areas

## Abstract

*Lycorma delicatula*, a globally invasive pest, has caused considerable economic losses in many countries. Determining the potential distribution range of *L. delicatula* is crucial for its effective management and control; however, our understanding of this species remains limited. In this study, Maxent model with occurrence records and environmental variables were fit first and then optimized by selecting the best combination of feature classes and regularization multipliers using the lowest score of corrected Akaike information criterion. Subsequently, we predicted global suitable areas for *L. delicatula* both currently and in the future (2041–2060, 2061–2080, and 2081–2100). The results indicated that the mean temperature of the driest quarter is the most important environmental variable limiting *L. delicatula* distribution. Currently, the suitable areas are concentrated in East Asia (mainly in China, South Korea, and Japan), central and eastern United States, and southern Europe. Compared with current environmental conditions, in all future climate scenarios, the number of suitable areas for *L. delicatula* increased. In addition, we revealed that suitable areas are likely to expand northward in the future. Our study results suggest that policymakers and governments should prioritize the development of pest management measures in suitable areas for *L. delicatula*, especially in high suitable areas, to control this invasive pest and minimize global economic losses.

## Graphical abstract

In future climate scenarios, the suitable areas for *L. delicatula* were larger than the current ones. Suitable areas are likely to expand northward in the future.

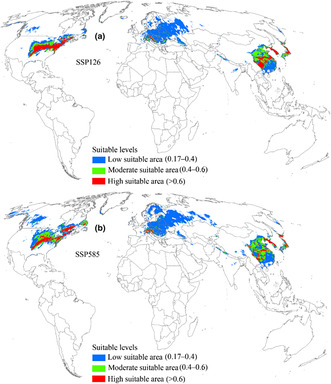

## 1 INTRODUCTION

Over the past two centuries, thousands of insect species have been transported worldwide (mostly unintentionally) and have colonized outside their native ranges (Liebhold et al., 2018). Invasive insects have significantly negatively affected agricultural productivity (Ziska et al., 2011), forest resources (Boyd et al., 2013), and human health (Juliano & Philip Lounibos, 2005), incurring considerable costs. It has been estimated that invasive insects cause global annual losses of at least $70 billion, with associated health costs exceeding $6.9 billion (Bradshaw et al., 2016). Various strategies have been implemented to control these invasive insects, showing varying degrees of effectiveness (McLaughlin & Dearden, 2019). The most effective strategy is to prevent their introduction (Gallien et al., 2012). Species distribution models are helpful to achieve this goal by determining their potential distribution under current and future climate conditions.

*Lycorma delicatula* (Hemiptera: Fulgoridae) is a planthopper species initially described in Shanxi, Shandong, and Hebei provinces in China (Liu, 1939). This pest has over 70 host plant species (Jung et al., 2022), such as black locusts (*Robinia pseudoacacia*), cottonwoods, willows (*Salix* spp.), grapes (*Vitis* spp.), and apples (*Malus* spp.) (Xiao, 1992; Zhang, 1993). This broad host range allows *L. delicatula* to infest various habitats, including agricultural, urban, suburban, and natural forested areas (Urban & Leach, 2023). Currently, *L. delicatula* has rapidly invaded many countries, including the United States (such as Maryland, New Jersey, Delaware, Virginia, and Ohio), Japan, and South Korea (Lee et al., 2019), and has become a highly notorious invasive pest. The nymphs and adults of *L. delicatula* frequently aggregate and harm plants directly by feeding on plant sap (Dara et al., 2015). Recent studies have shown that feeding by *L. delicatula* is most damaging to grapevines (Harner et al., 2022), but is less damaging than previously presumed to hardwood tree species (Hoover et al., 2023) and tree fruit (Nixon et al., 2023). For example, the damage to agriculture in the eastern United States occurred primarily in the vineyard system (Harner et al., 2022), resulting in strongly reduced yield (up to 90%), fruit quality, and sometime caused vine decline over multiple years (Urban, 2020). Moreover, a recent study predicted that *L. delicatula* have a high establish probability in the grape‐producing counties of California by 2033 in the western United States (Jones et al., 2022).

Numerous studies on *L. delicatula* have been conducted from different perspectives, including its biology (Keena & Nielsen, 2021; Kreitman et al., 2021), ethology (Keller et al., 2020; Leach & Leach, 2020), and control measures (Leach et al., 2019). These studies aimed to provide crucial information for controlling this pest. Notably, the basic condition for pest management is determining their potential distribution, which is currently not well understood for *L. delicatula*.

Species distribution models have become effective tools for projecting the distribution of invasive insects (Elith, 2017; Huang et al., 2019; Liu & Shi, 2020; Ramos et al., 2018). To the best of our knowledge, three studies have used these models to predict the global distribution of *L. delicatula*. Jung et al. (2017) identified potential high‐risk areas in the United States, Brazil, Mexico, Congo, China, Japan, and South Korea based on the CLIMEX model, but this study relied solely on distribution data from South Korea. Wakie et al. (2020) observed highly suitable areas in Asia, Oceania, South America, North America, Africa, and Europe using the Maxent model. Nevertheless, they did not optimize two key parameters (i.e., feature classes and regularization multipliers), which affected its accuracy (Phillips & Dudík, 2008) and the determination of the distribution range. Huron et al. (2022) also established the potential risk for the 50 U.S. states and 223 countries by the Maxent model. Notably, a common limitation from three studies aforementioned is that they do not predicted the global distribution range of *L. delicatula* under future climate change. The lack of knowledge about future distribution is not conducive to the management and control of pests, because the distribution of pests often shifts in the future relative to the current time, which makes that existing pest management strategies may become inadequate and require modification. Thus, designing an excellent species distribution model for forecasting the future global distribution of *L. delicatula* is urgent.

The Maxent model is based on a machine learning algorithm that estimates the distribution (geographic range) of a target species by determining the probability distribution of maximum entropy while considering constraints derived from environmental variables at several occurrences (Phillips et al., 2017). This model is the most commonly used species distribution model owing to its superior performance over other models (Merow et al., 2013; Nair & Peterson, 2023; Phillips et al., 2006; Zhou et al., 2023). Thus, in this study, the Maxent model was employed to predict the current and future potential suitable areas of *L. delicatula* globally. Our study mainly addresses two key questions: (1) Where are the current and future potential suitable areas located? (2) How will the potential suitable areas change under future climate conditions?

## 2 MATERIALS AND METHODS

### 2.1 Species distribution data

**FIGURE 1:**
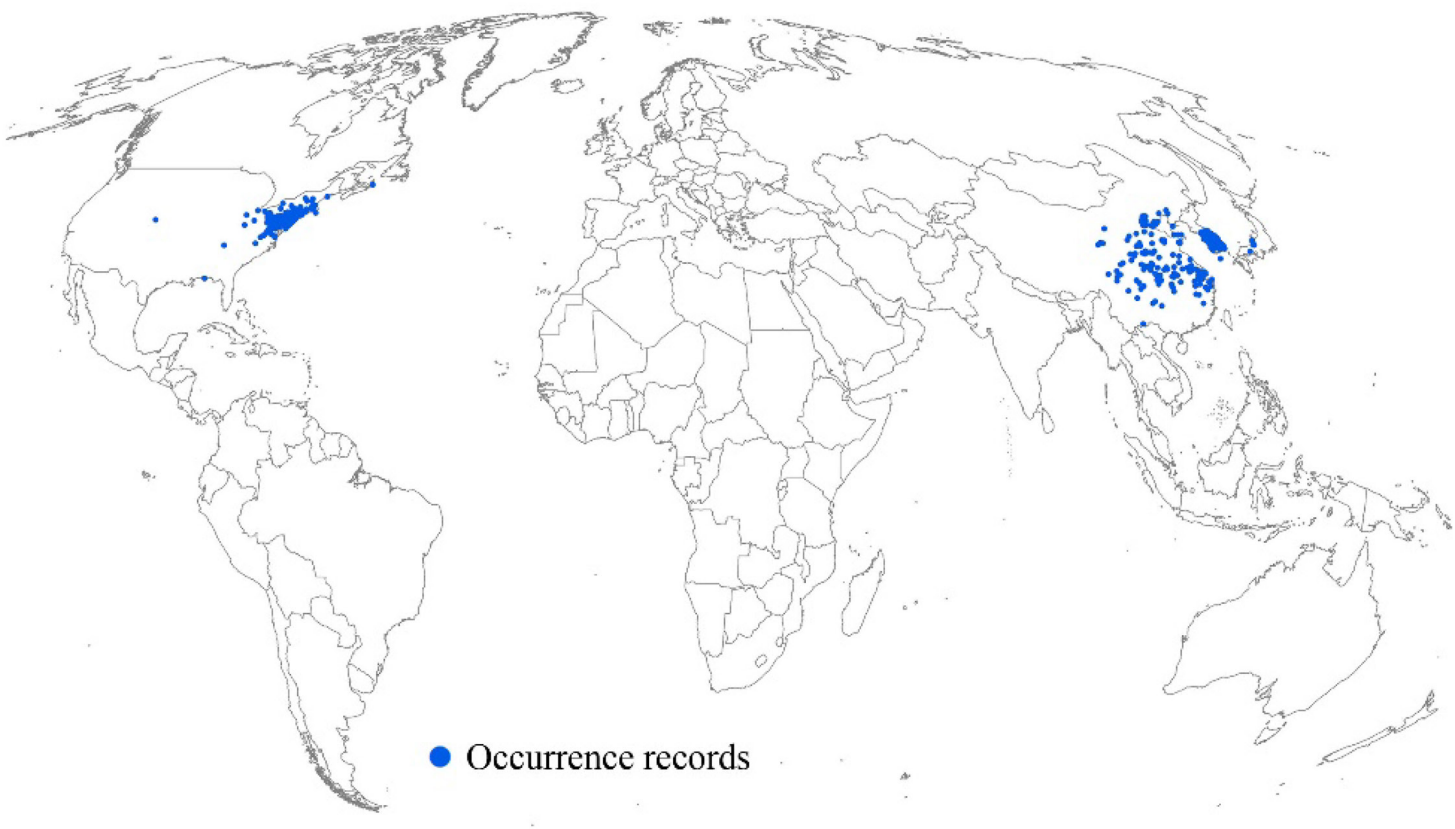
Global occurrence records of *L. delicatula*.

The 15,599 raw occurrence records of *L. delicatula* were collected from the literature and Global Biodiversity Information Facility (GBIF, 2023). The GBIF occurrence records with common errors in biological collections (e.g., sea coordinates and coordinate–country mismatches) and high coordinate uncertainty (>20 km) were cleaned using the CoordinateCleaner Package via R 4.2.1 (Zizka et al., 2019). Furthermore, to reduce the influence of sampling bias on our prediction results, the occurrence records were spatially thinned at a distance of 20 km, based on the spThin package via R 4.2.1 (Aiello‐Lammens et al., 2015). This process retains the maximum number of occurrence records for a given 20 km using a randomization approach. Consequently, 464 occurrence records were obtained globally, and they are distributed in China (e.g., Guizhou and Yunan provinces), South Korea, southern Japan, and the United States (e.g., North Carolina, Tennessee, and Virginia states) (Figure 1).

### 2.2 Environmental variables

The 19 bioclimatic variables (Bio1–Bio19) and altitude data were widely used to predict the suitable areas of insects in the species distribution models (e.g., Wei et al., 2018, 2019; Zhao et al., 2024a). Bioclimatic variables are related to temperature and precipitation, and represent annual trends (e.g., annual precipitation), seasonality (e.g., isothermality), and limiting factors (e.g., mean temperature of the wettest quarter and driest quarter) (WorldClim website, https://www.worldclim.org). In this study, 19 bioclimatic variables recorded from 1970 to 2000 were selected. To reduce collinearity among 20 environmental variables, we followed a specific process. First, environmental variable values from the occurrence records were extracted using ArcGIS 10.7. Second, pairwise Pearson correlation coefficients (*r*) were calculated among the environmental variables using SPSS 25. Only one variable was retained when the correlation coefficient of a pair of variables |*r*| was ≥0.85. Third, variance inflation factor (VIF) value were computed for the retained variables using the same software, and the variable with the highest VIF value was omitted until it was <5. Finally, five environmental variables were selected for subsequent analysis: isothermality (Bio3), mean temperature of the wettest quarter (Bio8), mean temperature of the driest quarter (Bio9), annual precipitation (Bio12), and altitude.

Future bioclimatic variables were derived from three timeframes (2041–2060, 2061–2080, and 2081–2100) based on data from the Coupled Model Intercomparison Project Phase 6 with two shared socioeconomic pathways (SSP126 and SSP585). To control uncertainty in future climate models, we averaged climate data from the CanESM5, IPSL‐CM6A‐LR, and MIROC6 models (Zhao et al., 2024a). Moreover, we used current altitude data for future predictions, which are expected to undergo minimal changes over the next few decades.

All environmental variables were downloaded from the WorldClim website (https://www.worldclim.org) and had a spatial resolution of 5 arc min.

### 2.3 Optimized Maxent model

The feature classes (FC) and regularization multipliers (RM) in the Maxent model have affected its performance (Phillips & Dudík, 2008; Radosavljevic et al., 2014), indicating that these two parameters should be optimized (Muscarella et al., 2014). A FC is a simple mathematical transformation of an environmental variable and determine the kinds of constraints allowed in a model (Velasco & González‐Salazar, 2019). More FC enables more flexible and complex fits to the observed data, but they may require more data (Merow et al., 2013; Phillips & Dudík, 2008). RM is a value that seeks to balance model fit and complexity by warranting that predicted values do not fit too exactly the empirical constraints of environmental variables (Phillips & Dudík, 2008; Velasco & González‐Salazar, 2019). Here, using the ENMeval package in R 4.2.1, we employed RM values ranging from 0.5 to 4 (increments of 0.5) and eight different FC combinations (L, LQ, LQH, LQHP, LQHPT, QHP, QHPT, and HPT; L = linear, Q = quadratic, H = hinge, P = product, and T = threshold) to select the model with the lowest score of corrected Akaike information criterion (Muscarella et al., 2014). Consequently, LQHPT for FC and 1 for RM values were obtained. Five replications with cross‐validation and randomly 10,000 background points located across the globe were selected to run the model, and the cloglog output format was used, as it is the most appropriate for estimating the probability of presence (Phillips et al., 2017).

Model performance was evaluated using the area under the receiver operating characteristic curve (AUC) and the true skill statistic (TSS), both of which are considered excellent when their values exceed 0.9 and 0.8, respectively (Ben Rais Lasram et al., 2010; Bogawski et al., 2019). The relative importance of the environmental variables was assessed using the jackknife test.

### 2.4 Changes in suitable areas

We applied the threshold of maximum training sensitivity plus specificity (0.17) to divide suitable/unsuitable areas. Suitable areas were further divided into three levels: low suitable area (0.17–0.4), moderate suitable area (0.4–0.6), and high suitable area (>0.6).

To describe the changes in suitable areas under different climatic conditions, we identified patterns of contraction, expansion, and no change under future conditions relative to those under the current conditions. This analysis was conducted using ArcGIS 10.7 with SDMtoolbox (Brown, 2014).

## 3 RESULTS

### 3.1 Model evaluation

All AUC and TSS values for each run of the Maxent model exceeded 0.9 (Table 1). Moreover, the mean AUC and TSS values of the five replicates were 0.9716 and 0.9188, respectively, indicating the excellent performance of the model established in this study (Table 1).

**TABLE 1:** AUC and TSS values for each run and the mean from the Maxent model.

Replications	AUC	TSS
1	0.9711	0.9076
2	0.9715	0.9379
3	0.9755	0.9219
4	0.9723	0.9117
5	0.968	0.9152
Mean	0.9716	0.9188

### 3.2 Importance of environmental variables

**FIGURE 2:**
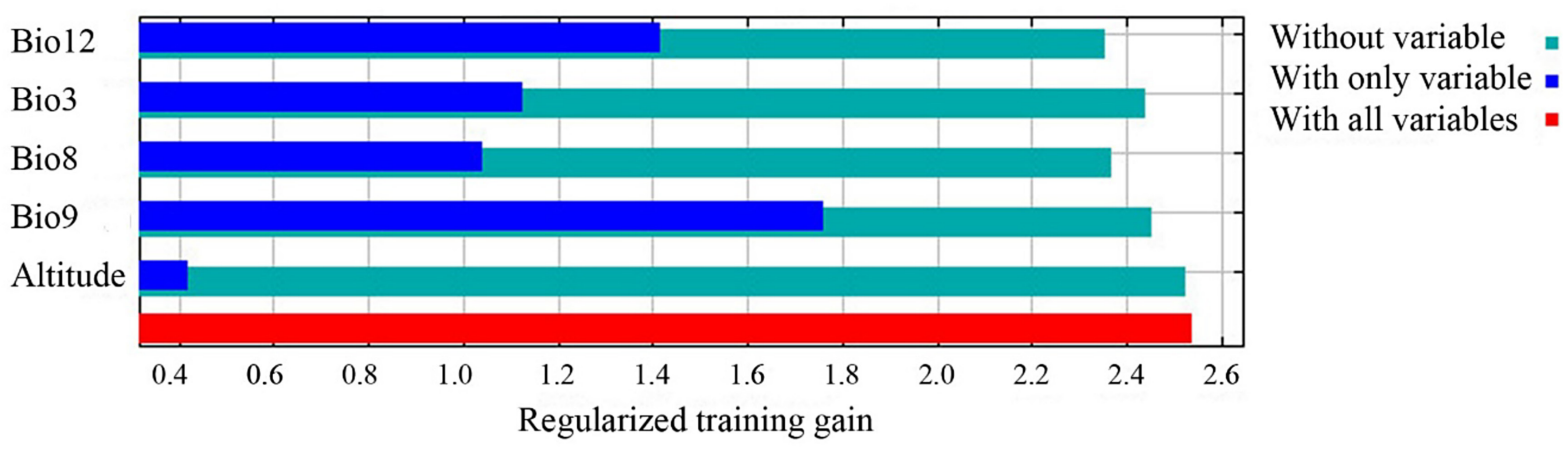
Environmental variable importance using the jackknife test.

The results of the jackknife test revealed relative importance of the five environmental variables in determining *L. delicatula* distribution (Figure 2). The mean temperature of the driest quarter (Bio9) emerged as the most important environmental variable, followed by annual precipitation (Bio12), isothermality (Bio3), and mean temperature of the wettest quarter (Bio8) (Figure 2). Evidently, altitude showed the lowest importance (Figure 2).

**FIGURE 3:**
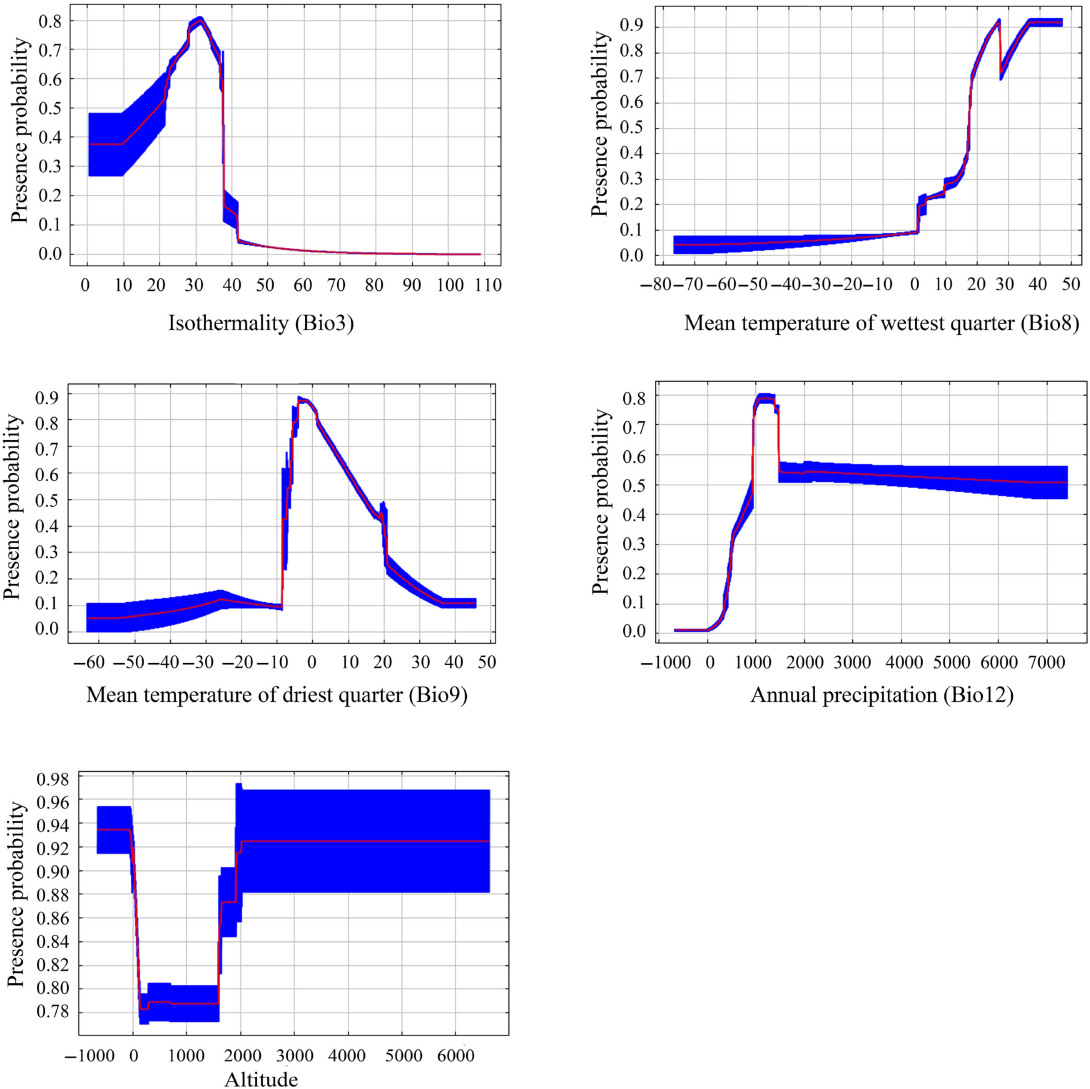
Response curves of the relationship between the presence probability of *L. delicatula* and five environmental variables.

Based on the response curves (Figure 3), the suitability of the environmental variables for *L. delicatula* was as follows: 0.48%–37.72% for isothermality (Bio3), 1.57–47.03°C for the mean temperature of the wettest quarter (Bio8), −8.38 to 28.49°C for the mean temperature of the driest quarter (Bio9), 426.15–7417.3 mm for annual precipitation (Bio12), and −651.7 to 6616.7 m for altitude.

### 3.3 Patterns and changes in suitable areas

**FIGURE 4:**
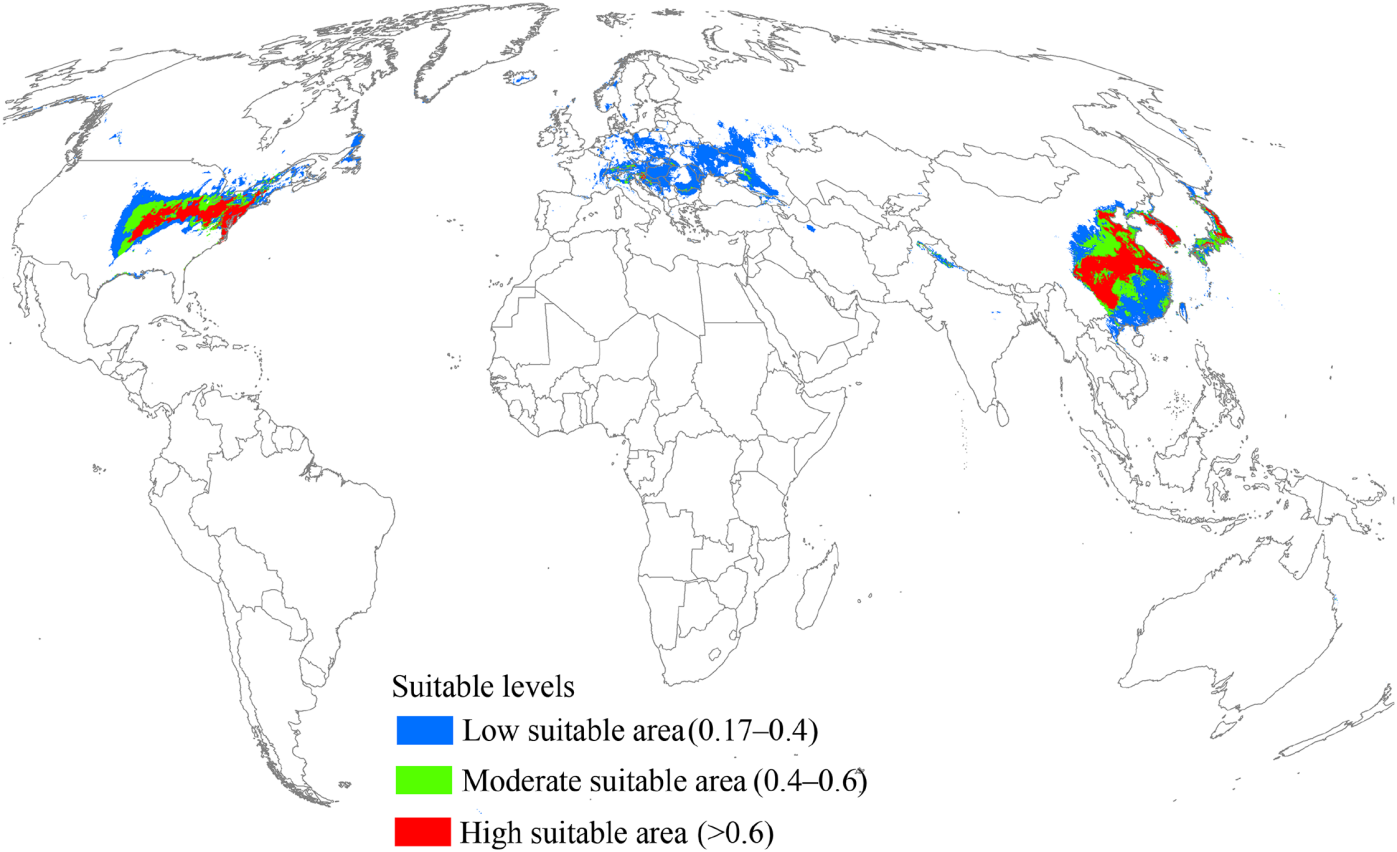
Currently suitable areas for *L. delicatula* on a global scale.

Under current environmental conditions, suitable areas for *L. delicatula* were mainly distributed in East Asia, central and eastern United States, and southern Europe (Figure 4), totaling an area of 7.89 × 10^6^ km^2^ (Table 2). Among them, the high, moderate, and low suitable regions covered areas of 1.89 × 10^6^, 1.62 × 10^6^, and 4.38 × 10^6^ km^2^, respectively (Table 2). In East Asia, suitable areas were mainly concentrated in China, South Korea, and Japan (Figure 4). The central and eastern United States were predicted to be highly suitable areas for *L. delicatula* (Figure 4). Conversely, in southern Europe, low suitable areas were dominant, with high and moderate suitable areas being minimal (Figure 4).

**TABLE 2:** Areas with suitability under current and future climatic conditions (km^2^).

Levels	Current	2041–2060		2061–2080		2081–2100	
		SSP126	SSP585	SSP126	SSP585	SSP126	SSP585
Total	7.89 × 10^6^	1.17 × 10^7^	1.24 × 10^7^	1.15 × 10^7^	1.4 × 10^7^	1.16 × 10^7^	1.64 × 10^7^
Low	4.38 × 10^6^	7.39 × 10^6^	8.19 × 10^6^	7.34 × 10^6^	9.7 × 10^6^	7.35 × 10^6^	1.19 × 10^7^
Moderate	1.62 × 10^6^	2.12 × 10^6^	2.20 × 10^6^	2.1 × 10^6^	2.6 × 10^6^	2.18 × 10^6^	3.21 × 10^6^
High	1.89 × 10^6^	2.10 × 10^6^	2.09 × 10^6^	2.13 × 10^6^	1.73 × 10^6^	2.09 × 10^6^	1.38 × 10^6^

**FIGURE 5:**
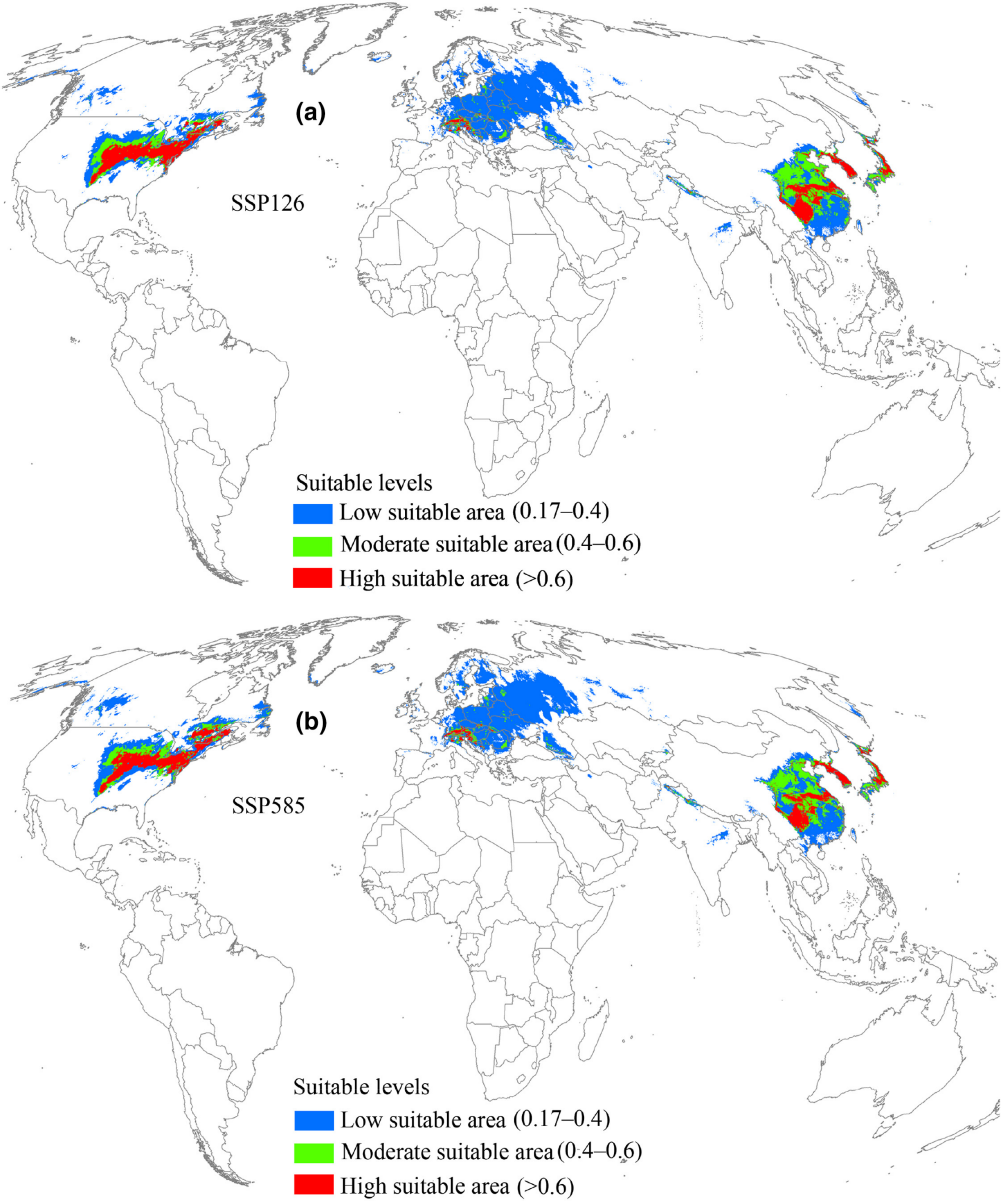
Suitable areas for *L. delicatula* during 2041–2060 on a global scale. SSP126 and SSP585 represent future climate scenarios for shared socioeconomic pathways 126 and 585, respectively.

**FIGURE 6:**
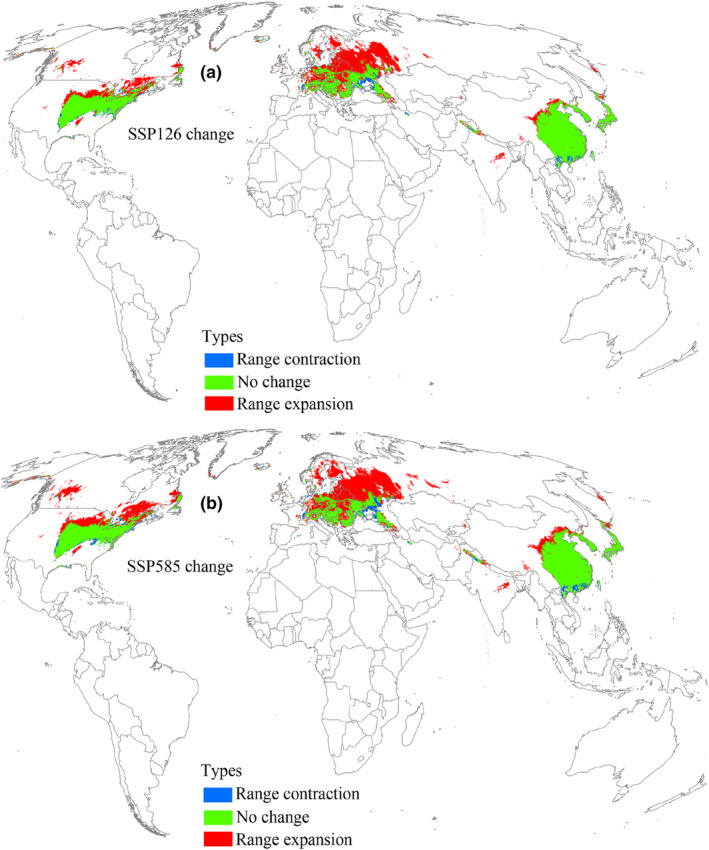
Suitable area changes for *L. delicatula* during 2041–2060 compared with the current environmental conditions. SSP126 and SSP585 represent future climate scenarios for shared socioeconomic pathways 126 and 585, respectively.

Under the 2041–2060 SSP126 scenario, suitable areas for *L. delicatula* were projected to cover an area of 1.17 × 10^7^ km^2^ (Table 2), indicating an increasing trend relative to current climatic conditions (Figure 5a). The high, low, and moderate suitable areas also increased consistently (Table 2). Under the SSP126 scenario of 2041–2060, the range expansion region reached an area of 4.51 × 10^6^ km^2^ (Table 3) and was concentrated in the north of suitable areas, mainly distributed in China, Europe, the United States, and Canada (Figure 6a). The region with no change in suitability covered the largest area at 7.1 × 10^6^ km^2^ (Table 3). Additionally, the range contraction region covered a limited area of 4.85 × 10^5^ km^2^ (Table 3). According to the 2041–2060 SSP585 scenario, suitable areas for *L. delicatula* covered an area of 1.24 × 10^7^ km^2^ (Table 2), which was greater than that under current conditions (Figure 5b). Compared with the SSP126 scenario, the areas of regions with range expansion and contraction were greater (Figure 6b), whereas the area of region with no change decreased (Table 3).

**TABLE 3:** Changes in suitable areas under future conditions compared with the current conditions (km^2^).

Types	2041–2060		2061–2080		2081–2100	
	SSP126	SSP585	SSP126	SSP585	SSP126	SSP585
Range expansion	4.51 × 10^6^	5.54 × 10^6^	4.52 × 10^6^	7.62 × 10^6^	4.5 × 10^6^	1.06 × 10^7^
Range contraction	4.85 × 10^5^	6.51 × 10^5^	5.4 × 10^5^	1.17 × 10^6^	4.7 × 10^5^	1.73 × 10^6^
No change	7.1 × 10^6^	6.94 × 10^6^	7.05 × 10^6^	6.42 × 10^6^	7.12 × 10^6^	5.86 × 10^6^

**FIGURE 7:**
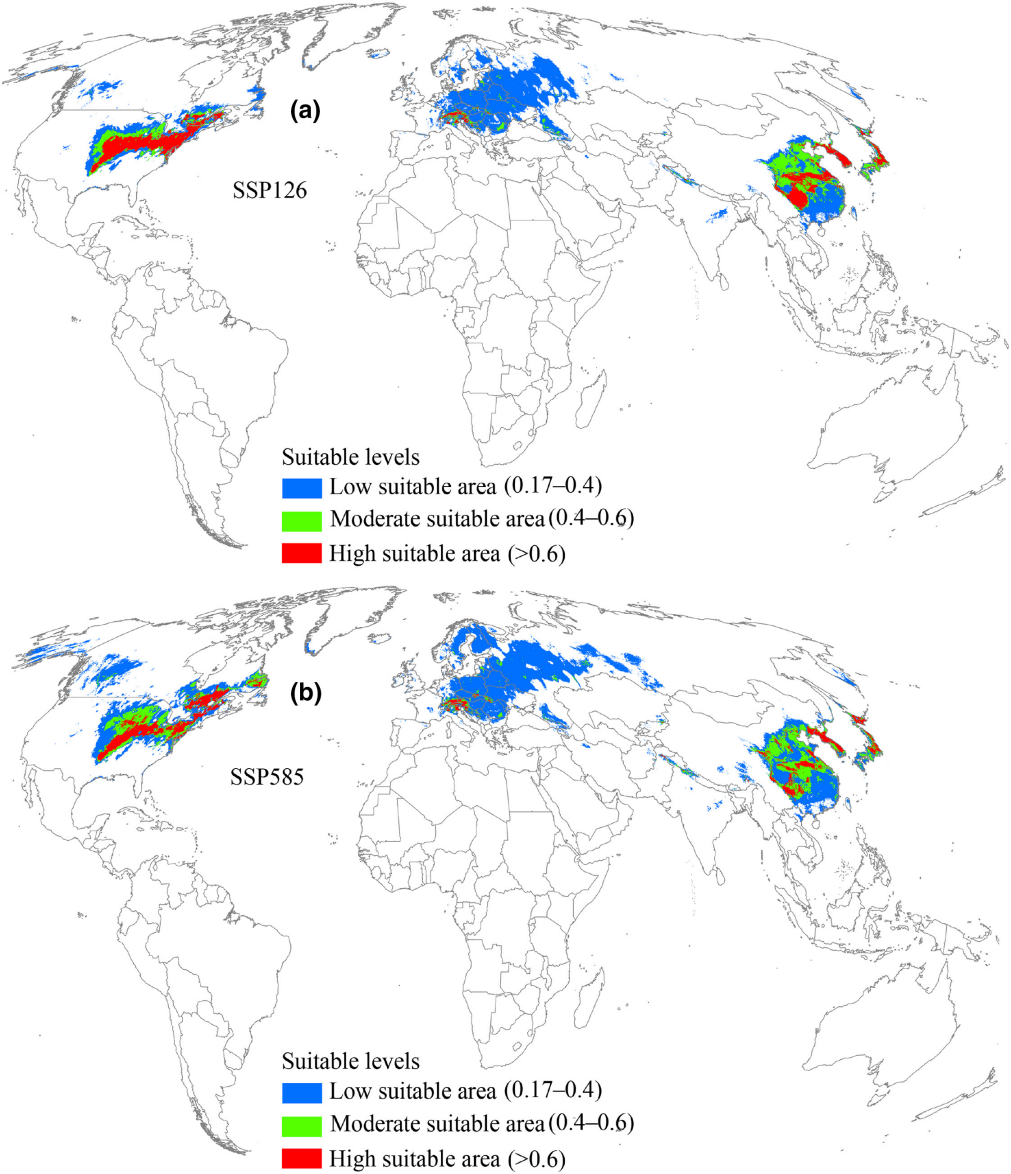
Suitable areas for *L. delicatula* during 2061–2080 on a global scale. SSP126 and SSP585 represent future climate scenarios for shared socioeconomic pathways 126 and 585, respectively.

**FIGURE 8:**
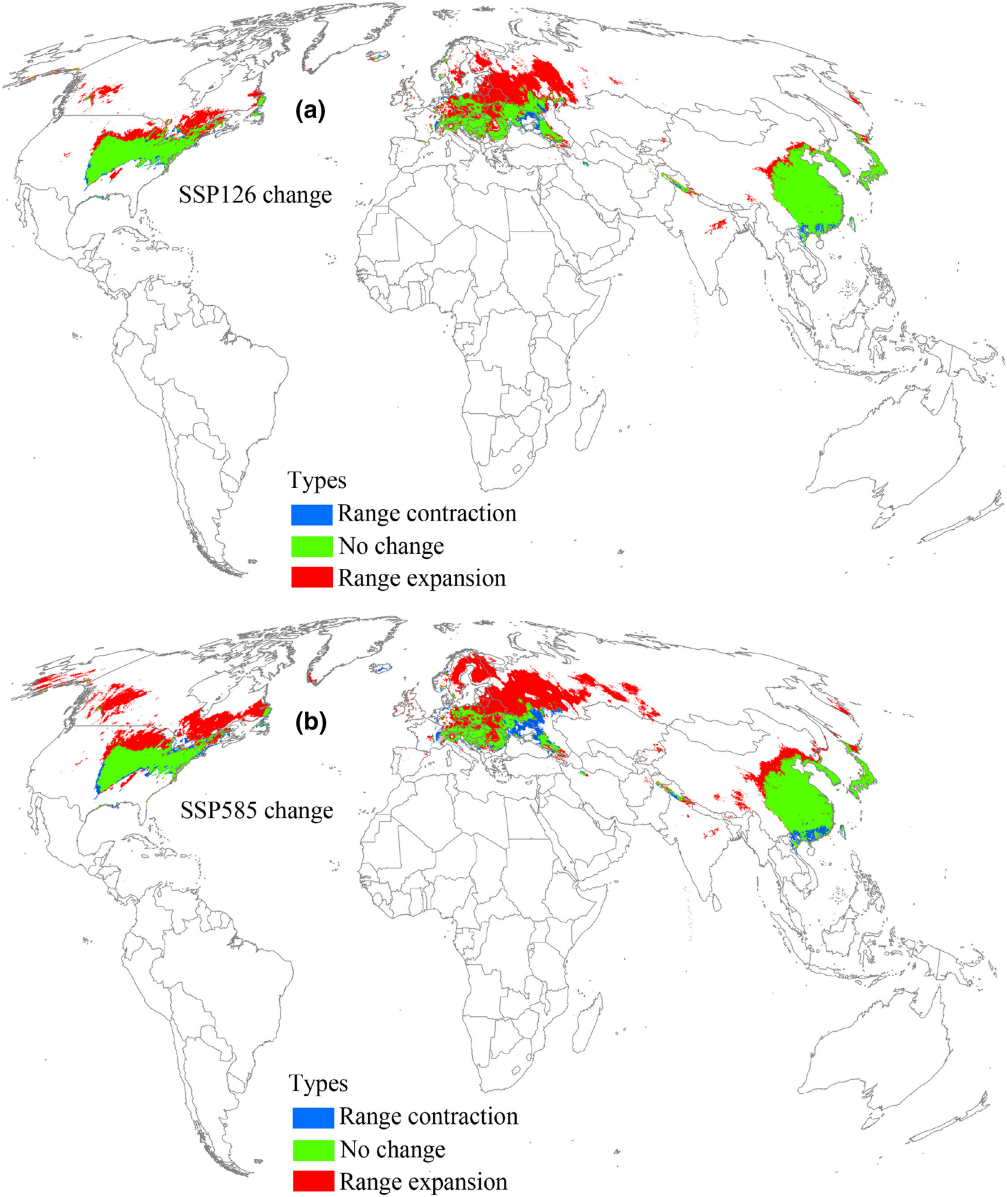
Suitable area changes for *L. delicatula* during 2061–2080 compared with the current environmental conditions. SSP126 and SSP585 represent future climate scenarios for shared socioeconomic pathways 126 and 585, respectively.

Under the 2061–2080 SSP126 scenario, the distribution pattern of suitable areas and the three different suitability levels were expected to increase compared to the current conditions (Figure 7a, Table 2). Similar to the two 2041–2060 scenarios, suitable areas expanded primarily toward the north (Figure 8a). Notably, in the SSP585 scenario, suitable area was predicted to occupy a larger distribution range relative to SSP126 scenario (Figure 7b), and the expansion of suitable areas into the north increased compared to the SSP585 scenario for 2041–2060 (Figure 8b). In this timeframe, the expansion, contraction, and no change areas covered 7.62 × 10^6^ km^2^, 1.17 × 10^6^ km^2^, and 6.42 × 10^6^ km^2^, respectively (Table 3).

**FIGURE 9:**
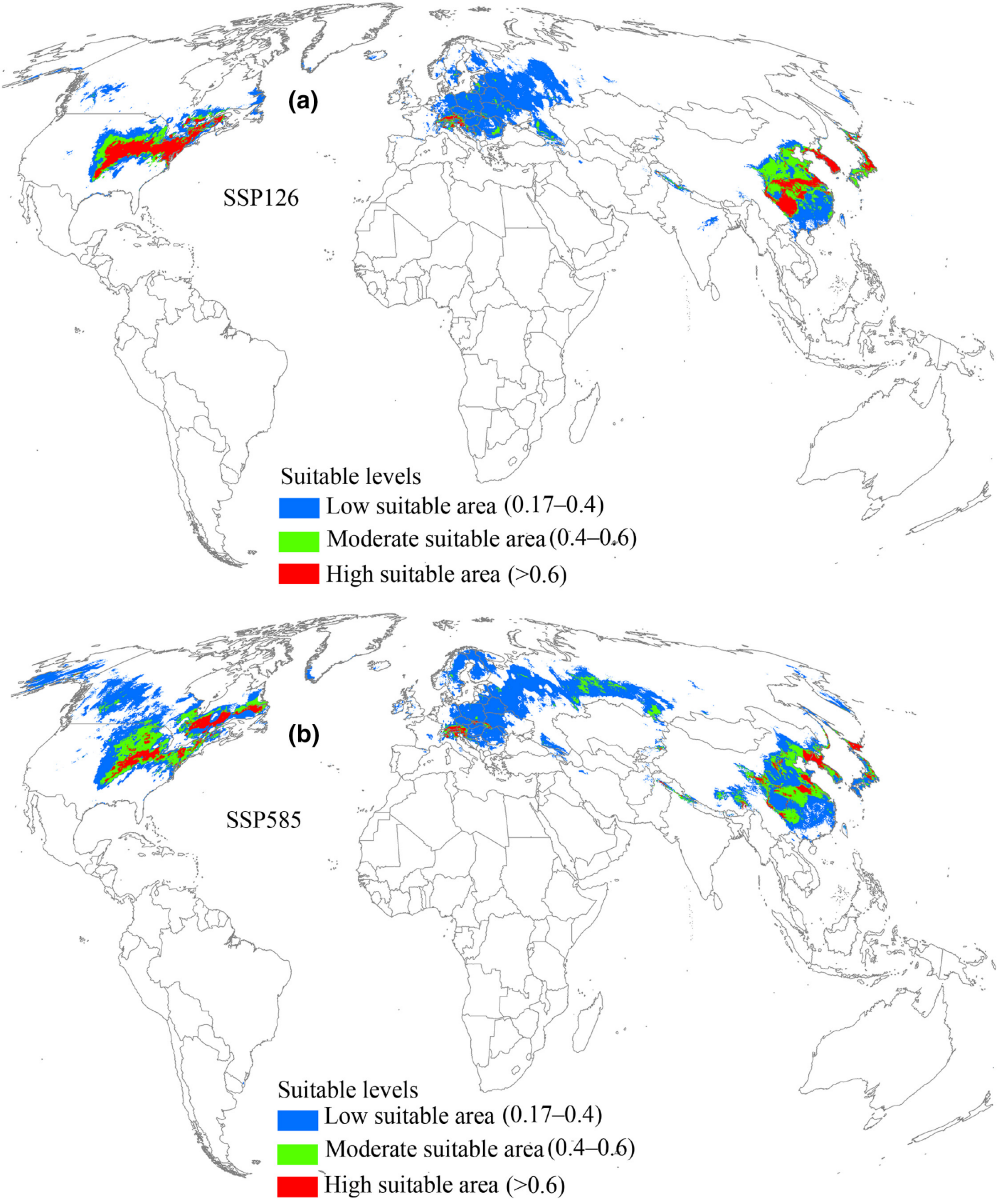
Suitable areas for *L. delicatula* during 2081–2100 on a global scale. SSP126 and SSP585 represent future climate scenarios for shared socioeconomic pathways 126 and 585, respectively.

**FIGURE 10:**
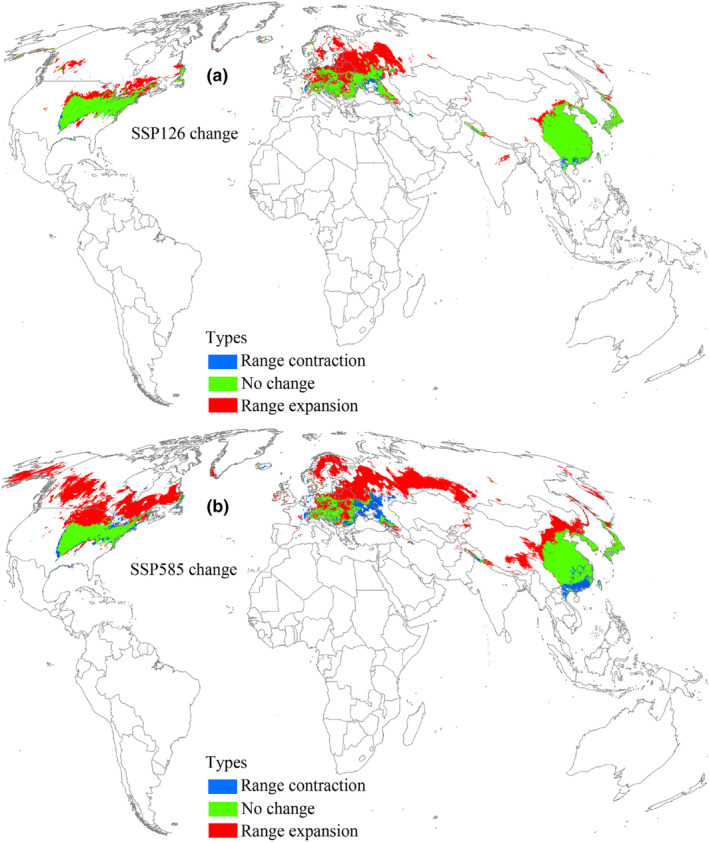
Suitable area changes for *L. delicatula* during 2081–2100 compared with the current environmental conditions. SSP126 and SSP585 represent future climate scenarios for shared socioeconomic pathways 126 and 585, respectively.

Under the 2081–2100 SSP126 scenario, suitable areas for *L. delicatula* were projected to increase compared with the current environmental conditions (Figure 9a) and reach 1.16 × 10^7^ km^2^ (Table 2), with high suitable areas predicted to cover 2.09 × 10^6^ km^2^ (Table 2). The increasing suitable areas were predicted to span 4.5 × 10^6^ km^2^ (Table 3), primarily expanding to the north (Figure 10a). Areas with no change and contraction measured 7.12 × 10^6^ km^2^ and 4.7 × 10^5^ km^2^, respectively (Table 3). Under the SSP585 scenario, the projected suitable areas reached a maximum value (1.64 × 10^7^ km^2^) in all future climate scenarios, whereas high suitable areas become the least (Figure 9b, Table 2). Notably, the suitable areas expanding northward exhibited the widest geographic range (Figure 10b), substantially surpassing those of other future climate scenarios.

## 4 DISCUSSION

Based on occurrence records and environmental variables, we developed an optimized Maxent model to predict current and future global suitable areas for *L. delicatula* and further explore changes in these suitable areas. Two evaluation metrics (AUC and TSS) consistently demonstrated the exceptional performance of the model.

The model results showed that the mean temperature of the driest quarter (Bio9) was the most important environmental variable influencing *L. delicatula* distribution. The importance of this variable was demonstrated in the distribution of this pest in the United States using the Maxent model (Wakie et al., 2020). Consistent with previous studies on distribution modeling of other insects (Abou‐Shaara et al., 2021; Amaro et al., 2022; Wei et al., 2018), temperature‐related variables were more important than precipitation‐related variables. This could be attributed to insects being poikilotherms, with temperature strongly affecting their physiological activities (Zhang et al., 2023) and thus predominantly determining their geographical range. In addition, it is crucial to acknowledge that altitude variables undeniably influence insect distribution, as shown in previous studies (Schuldt & Assmann, 2009, 2011). However, in this study, the effect of altitude on *L. delicatula* distribution is lowest in all environmental variables.

The most important economic crops damaged by *L. delicatula* is grapevines. California grows more grapes than any other state in the United States and produces 82% of the national grape crop. Several previous studies have predicted that California is suitable for *L. delicatula* to survive in the current and future (Jung et al., 2017; Jones et al., 2022; Wakie et al., 2020), whereas our predictions results showed that California is not suitable. Thus, this suggests that concerns about *L. delicatula* being able to damage California grapevines may be overdone. In addition, previous research results also show that the top five grape‐growing Europe countries including Spain, France, Italy, Portugal, and Romania are also environmentally suitable for *L. delicatula* (Jung et al., 2017; Wakie et al., 2020). In our study, suitable areas were not found in Portugal, and the remaining four countries had smaller range of suitable areas. However, the prediction results further indicated that these suitable areas will exist in the future, suggesting that the potential negative economic impact of *L. delicatula* on grape production is permanent. It is worth noting that Wakie's study (Wakie et al., 2020) also used the Maxent model, but did not strictly optimize the combinations of FC and RM that seriously affect the model performance and prediction results; however, we did. In this case, suitable areas predicted by our study are more accurate.

Numerous studies have demonstrated that pest distributions can either expand or contract under the influence of climate change (Lee et al., 2021; Ramasamy et al., 2021; Wang et al., 2021; Wang et al., 2020; Xu et al., 2020; Zhao et al., 2024b). In our study, we observed that the suitable habitat for *L. delicatula* is expected to expand significantly (mainly northward) in the future, indicating increased invasive potential. One typical region affected by this expansion is Europe. Under current climate conditions, suitable areas appear only in southern Europe. However, under all future climate scenarios, especially the SSP585 scenarios  for 2061–2080 and 2081–2100, suitable areas are projected to have expanded into northern Europe. This expansion may be due to the region becoming warmer in the future, according to our findings that temperature is the main factor affecting *L. delicatula* distribution (Figure 2) and the occurrence rate in high‐temperature conditions is expected to increase (Figure 3). Additionally, we observed that the high suitable areas did not consistently increase in the future compared to the present, which aligns with findings from previous studies (e.g., Gao et al., 2022; Wei et al., 2019). Enhanced management strategies are essential for regions with high suitable habitats, such as the central and eastern United States. Furthermore, we noted that the suitable areas for *L. delicatula* under the SSP585 scenario consistently exceeded those under the SSP126 scenario. Since SSP585 represents a high‐forcing scenario and SPP126 represents a low‐forcing scenario, the former predicts higher temperatures in the future. Consequently, our results are consistent with the understanding that warmer temperatures increase opportunities for *L. delicatula* population establishment (Figure 3).

The suitable areas determined in this study are valuable for developing pest management strategies to effectively control *L. delicatula*. First, policymakers and governments should prioritize substantial efforts to manage *L. delicatula* in its current and future suitable areas, especially in highly suitable areas. Second, a range of appropriate and sustained proactiveness management strategies, such as monitoring and strict quarantine measures, should be established in infested areas. Control measures must be promptly developed, and this pest should be eliminated upon discovery.

A common assumption in species distribution models is that species distributions are in equilibrium with environments (i.e., species occur in all climatically suitable areas). For invasive species, the invasion process may not be complete; therefore, such species may not have occupied all suitable environments (Wilson et al., 2007). Consequently, the modeling for invasive species may be biased (Broennimann & Guisan, 2008). To solve this problem, a classical approach is to train the models on native distributional areas (i.e., only using native occurrence records) because these areas were considered to have the advantage of achieving a higher probability of distributional equilibrium (Jiménez‐Valverde et al., 2011). Nevertheless, although the inclusion of invasion records in species distribution models is controversial, it is widely argued that these records can better approximate future invasion potential (Morey & Venette, 2020). Most importantly, recent studies have shown that models using occurrence records from both native and invasive areas accurately predict potential distribution of invasive species (e.g., Etges et al., 2023; Magory, 2019). Thus, the global potential distribution of *L. delicatula* predicted by this study model is still valuable.

Species distribution is influenced by a combination of biotic and abiotic factors, and incorporating both factors into species distribution models can improve the accuracy of the model (de Araújo et al., 2014; Giannini et al., 2013; Lippitt et al., 2008). Unfortunately, in this study, we did not consider a small range of biotic factors that influence *L. delicatula* distribution when constructing the Maxent model. Host plants influence the geographical range of *L. delicatula*, as demonstrated by a recent study by Jung et al. (2022). This study reported that tree height and diameter at the root collar of *Ailanthus altissima* trees, one of the main host plants for *L. delicatula*, significantly increased the number of egg masses in this pest, and thus *Ailanthus altissima* trees favor population growth and increases invasion risk. Additionally, studies have shown that human activity increases the risk of *L. delicatula* invasion (Cook et al., 2021; Ladin et al., 2023). Therefore, for more accurate predictions in the future, host plants and human activity variables such as the human influence index should be included as predictor variables in species distribution models.

## 5 CONCLUSIONS

We developed an optimized Maxent model to predict the current and future suitable areas for the invasive pest *L. delicatula* worldwide. Our model results predicted that East Asia, central and eastern United States, and southern Europe are the main suitable areas under current environmental conditions. However, in future climate scenarios, the suitable areas for *L. delicatula* were larger than the current ones, indicating a significant increase in the invasive potential of this pest. Furthermore, our results indicated a northward expansion trend of suitable areas. This study provides valuable insights into the risk of introducing *L. delicatula* under both current and future conditions and can be used to develop appropriate pest management strategies.

## AUTHOR CONTRIBUTIONS

**Zhengxue Zhao:** Conceptualization (lead); formal analysis (lead); visualization (lead); writing – original draft (lead). **Lin Yang:** Conceptualization (supporting); data curation (lead); formal analysis (supporting). **Xiangsheng Chen:** Methodology (lead); visualization (supporting); writing – original draft (supporting).

## FUNDING INFORMATION

This research was funded by the National Natural Science Foundation of China (No. 31860209) and the Science and Technology Support Program of Guizhou Province (No. 20201Y129).

## CONFLICT OF INTEREST STATEMENT

We declare no conflict of interest in connection with the work submitted.

## Data Availability

Raw distribution data, environment variables, and R code are available in figshare: https://doi.org/10.6084/m9.figshare.24460603.
